# Comparing the Intramedullary Nail and Extramedullary Fixation in Treatment of Unstable Intertrochanteric Fractures

**DOI:** 10.1038/s41598-018-20717-2

**Published:** 2018-02-02

**Authors:** Wen-Qiang Zhang, Jian Sun, Chun-Yu Liu, Hong-Yao Zhao, Yi-Feng Sun

**Affiliations:** 1 Orthopaedics Department of Shandong Provincial Qianfoshan Hospital, Shandong University, Jingshi Road 16766, Jinan, Shandong 250014 PR China; 2 Community health service center of zhanhua fuyuan street, Yanhe road 453, Binzhou, Shandong 256800 PR China

## Abstract

Treatment options for unstable intertrochanteric fractures include intramedullary nail and extramedullary fixation, although evidence regarding the most appropriate treatment for such fractures remains controversial. Our hypothesis was that there would be no obvious differences in mortality rates, functional outcomes and complications between the two groups. We therefore conducted a meta-analysis to compare the relative advantages of intramedullary nail and extramedullary fixation. A total of 10 randomized controlled trials including only patients with unstable intertrochanteric fractures were included in the final analysis. We found that no statistically significant difference in one-year mortality was observed between the two groups (RR: 0.78, 95% CI: 0.55–1.10, p = 0.160). Analysis of exact *p* values from five included studies indicated that functional outcomes were markedly better for patients of the intramedullary nail group when compared with those of the extramedullary fixation group (*p* = 0.0028), although evidence remains controversial. Higher incidences of all complications were noted for extramedullary fixation (RR:1.48, 95% CI: 1.12–1.96, *p* = 0.006). However, no significant differences in implant-related complications were observed between the two groups (RR:1.20, 95% CI: 0.73–1.97, *p* = 0.475). Therefore, comparing with extramedullary fixation, the intramedullary nail method would be more reliable and should be encouraging for unstable intertrochanteric fractures.

## Introduction

Intertrochanteric hip fractures have become increasingly common, most frequently occurring in older adults. Between 35–40% of these fractures are classified as unstable (AO/ASIF classification: 31-A2/31-A3) and are thus associated with high rates of morbidity and mortality^1^. Management of unstable intertrochanteric fractures remains challenging, particularly regarding the improvement of mobility and functional outcomes^2,3^. Modern treatment options for unstable intertrochanteric fractures include intramedullary (e.g., PFNA, Proximal femoral nail antirotation; PFN, Proximal femoral nail; IMHS, Intramedullary hip screw; TN, InterTan nail; GN, Gamma nail) and extramedullary (e.g., DHS, Dynamic hip screw; CHS, Compression hip screw; PFLCP, proximal femoral locking compression plate; AMBI, AMBI sliding screw; SHS, Sliding hip screw) fixation, both of which have received empirical support^4^. Initially, the extramedullary sliding screw (e.g., DHS) was considered standard in the acute management of intertrochanteric fractures, though the use of intramedullary devices gradually increased, surpassing that of extramedullary devices in 2008^5,6^. Several studies have suggested that intramedullary devices may be the more effective option for internal fixation of unstable intertrochanteric femoral fractures, and that extramedullary fixation should be implemented with caution due to higher complication rates and poorer functional outcomes. However, other studies have reported no significant differences in outcomes between intramedullary nail and extramedullary fixation^7–10^. Furthermore, most previous studies have been retrospective and/or non-specific for unstable intertrochanteric fractures, necessitating further investigation^11–15^. Therefore, we conducted a meta-analysis of 10 randomized controlled trials involving only patients with unstable intertrochanteric fractures in order to compare mortality rates, functional outcomes and complications between intramedullary nail and extramedullary fixation procedures, and our hypothesis was that there would be no obvious differences in mortality rates, functional outcomes and complications between the two groups.

## Results

### Study characteristics

**Figure 1:**
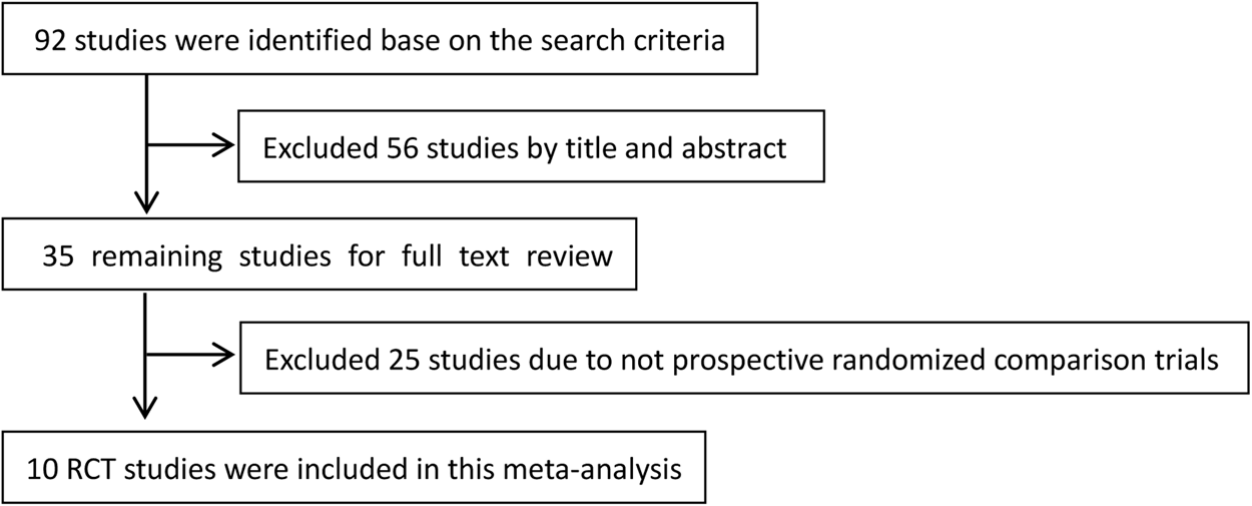
Flow diagram depicting the study selection procedure. A total of 10 RCTs involving 1,277 patients were included in the final meta-analysis.

We initially identified 92 studies via our search of the PubMed, Embase, Web of Science, and CBM databases. A total of 56 reports did not meet the inclusion criteria and were excluded following review of the title and abstract. Of the 35 remaining studies that underwent a full-text review, 25 were excluded because they were not randomized controlled trials. A total of 10 Randomised Controlled Ttrials (RCTs) involving 1,277 patients were included in the final meta-analysis. The study flow diagram is presented in Fig. 1^16–25^.

### Meta -analysis results

**Figure 2:**
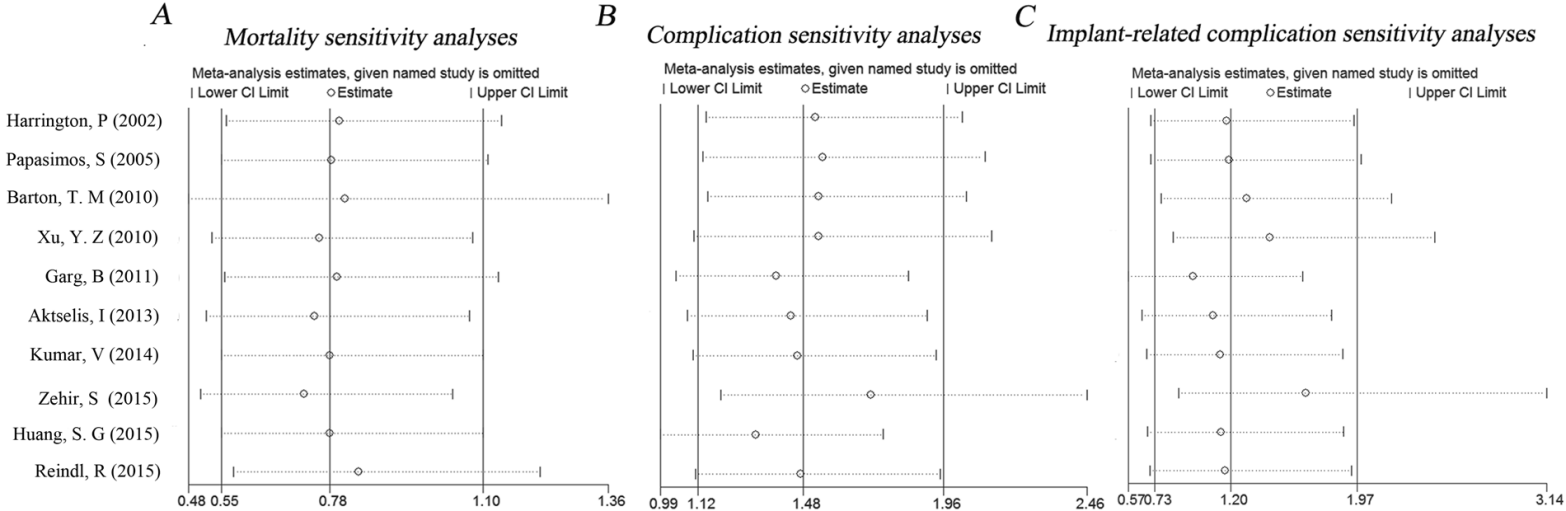
Sensitivity analyses of mortality, complications, and implant-related complications. The results indicated that all studies were stable.

Table 1 summarized the main characteristics of the 10 included studies. Table 2 was used to depict the Heterogeneit analysis and Forest plot results of Mortality, Function, Complications, Implant-related complications. Sensitivity analyses of mortality, complications, and implant-related complications were performed to assess the stability of the pooled effects. Our results indicated that all studies were stable (Fig. 2).

**Table 1:** Main characteristics of all articles included in the meta-analysis.

Order	Study	Published Year	Country	Method	No.	Function scoring system	P Value	Mortality	Compli- cation	Implant-related complication
1	Harrington, P. *et al*.^23^	2002	UK	CHS IMHS	52 50	Living and ambulatory status	>0.05	2 4	2 3	2 1
2	Papasimos, S. *et al*.^21^	2005	Greece	DHS GN, PFN	40 80	Salvati and Wilson scoring system	>0.05	1 3	10 17	2 3
3	Barton, T. M. *et al.*^17^	2010	UK	SHS GN	110 100	EuroQol 5D	>0.05	24 32	2 3	2 3
4	Xu, Y. Z. *et al*.^16^	2010	China	DHS PFNA	55 51	Mobility score	0.0146	3 2	21 15	1 5
5	Garg, B. *et al*.^22^	2011	India	DHS PFNA	39 42	Harris hip score	<0.05	2 4	6 0	6 0
6	Aktselis, I. *et al*.^18^	2013	Greece	AMBI GN	40 40	Barthel Index	0.036	5 4	3 0	3 0
8	Zehir, S. *et al*.^20^	2015	Turkey	DHS PFN	102 96	Walking ability	0.14	5 2	29 25	8 12
9	Huang, S. G. *et al*.^19^	2015	China	DHS, PFLCP PFNA	60 30	Harris hip score	0.06	0 0	21 2	3 0
10	Reindl, R. *et al*.^25^	2015	Canada	DHS ITN, GN	92 112	LowerEx-tremity Measure (LEM)	0.69	6 13	2 1	2 1

Abbreviations: PFNA, Proximal femoral nail antirotation; DHS, Dynamic hip screw; PFN, Proximal femoral nail; CHS, Compression hip screw; IMHS, Intramedullary hip screw; PFLCP, proximal femoral locking compression plate; AMBI, AMBI sliding screw; SHS, Sliding hip screw; ITN, InterTan nail; GN, Gamma nail.

**Table 2:** Heterogeneit analysis and Forest plot results of Mortality, Function, Complications, Implant-related Complications.

Data Results	Heterogeneit analysis			Forest plot result		
	^χ2^	P	I^2^	RR	95% CI	P value
Mortality	4.01	0.778	0.00%	0.78	0.55–1.10	0.160
Function	—	—	—	—	—	0.0028
Complications	11.36	0.252	20.8%,	1.48	1.12–1.96	0.006
Implant-related complications	11.73	0.229	23.3%	1.20,	0.73–1.97	0.475

### Mortality

**Figure 3:**
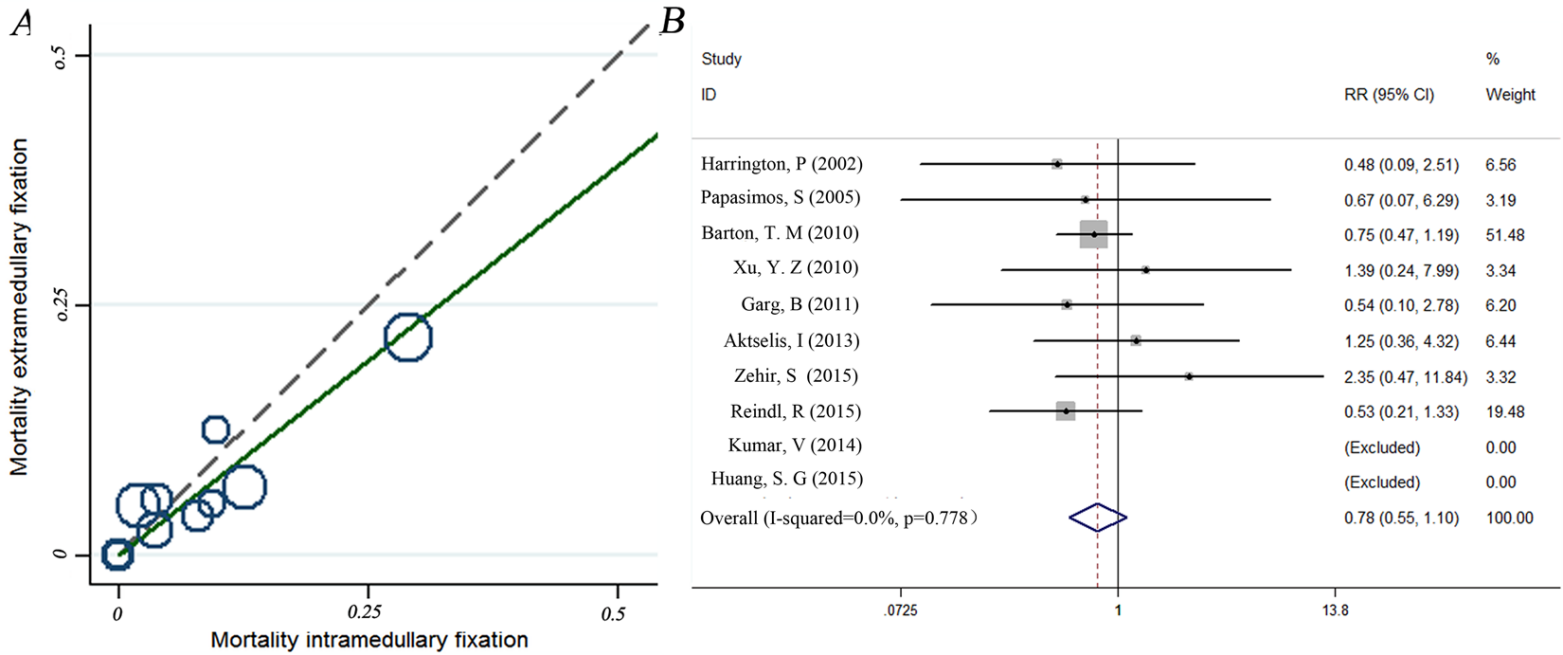
L’Abbé plot (**A**) and forest plot (**B**) for the meta-analysis of mortality between intramedullary nail and extramedullary fixation. L’Abbé analyses indicated no statistical heterogeneity (χ^2^ = 4.01,P = 0.778, I2 = 0.00%), and data pooled using a fixed-effects model indicated no statistically significant difference between the two groups (RR:0.78, 95% CI: 0.55–1.10, p = 0.160).

The incidence of mortality was reported in all 10 studies. Five studies^17,21–23,25^ reported higher mortality for the extramedullary fixation group than the intramedullary nails group, although the opposite result was noted in three other studies^16,18,20^. No deaths were reported in the remaining two studies, which were excluded from this portion of analysis^19,24^. Chi-square, I-square, and L’Abbé analyses indicated no statistical heterogeneity (χ^2^ = 4.01,P = 0.778, I^2^ = 0.00%) (Fig. 3A), and data pooled using a fixed-effects model indicated no statistically significant difference between the two groups (RR:0.78, 95% CI: 0.55–1.10, *p* = 0.160,) (Fig. 3B).

### Function

Seven primary functional scoring systems were utilized among the 10 included studies: Lower Extremity Measure (LEM), Salvati and Wilson Scoring System (SWS), Ambulatory status and Living situation, Harris Hip Score (HHS), Walking ability, EuroQol 5D, Mobility score. Barthel Index, Functional Independence Measure (FIM), Timed “Up & Go” (TUG) test (measuring the time needed to rise from a sitting position and walk 20 m), and timed two-minute walk test scores were also evaluated in some studies^26,27^. Exact *p* values were reported in five of the included studies^16,18,20,21,25^, while the remaining studies only specified whether results were statistically significant. Functional outcomes were markedly better in the extramedullary fixation group than in the intramedullary nail group in three studies^16,18,22^. A P-value <0.05 was considered statistically significant in seven studies^17,19–21,23–25^. Given that there is no universal functional scoring system for measuring postoperative function and the limited number of exact *p* values, only five studies were included in this portion of the meta-analysis. Significant differences were noted between the intramedullary nail and extramedullary treatment groups of these studies (p = 0.0028), although these results remain questionable, as four of the five remaining studies reported no significant difference between the two groups. Therefore, it is necessary to establish a universal system for the assessment of postoperative function in patients with unstable intertrochanteric fractures.

### Complications

**Figure 4:**
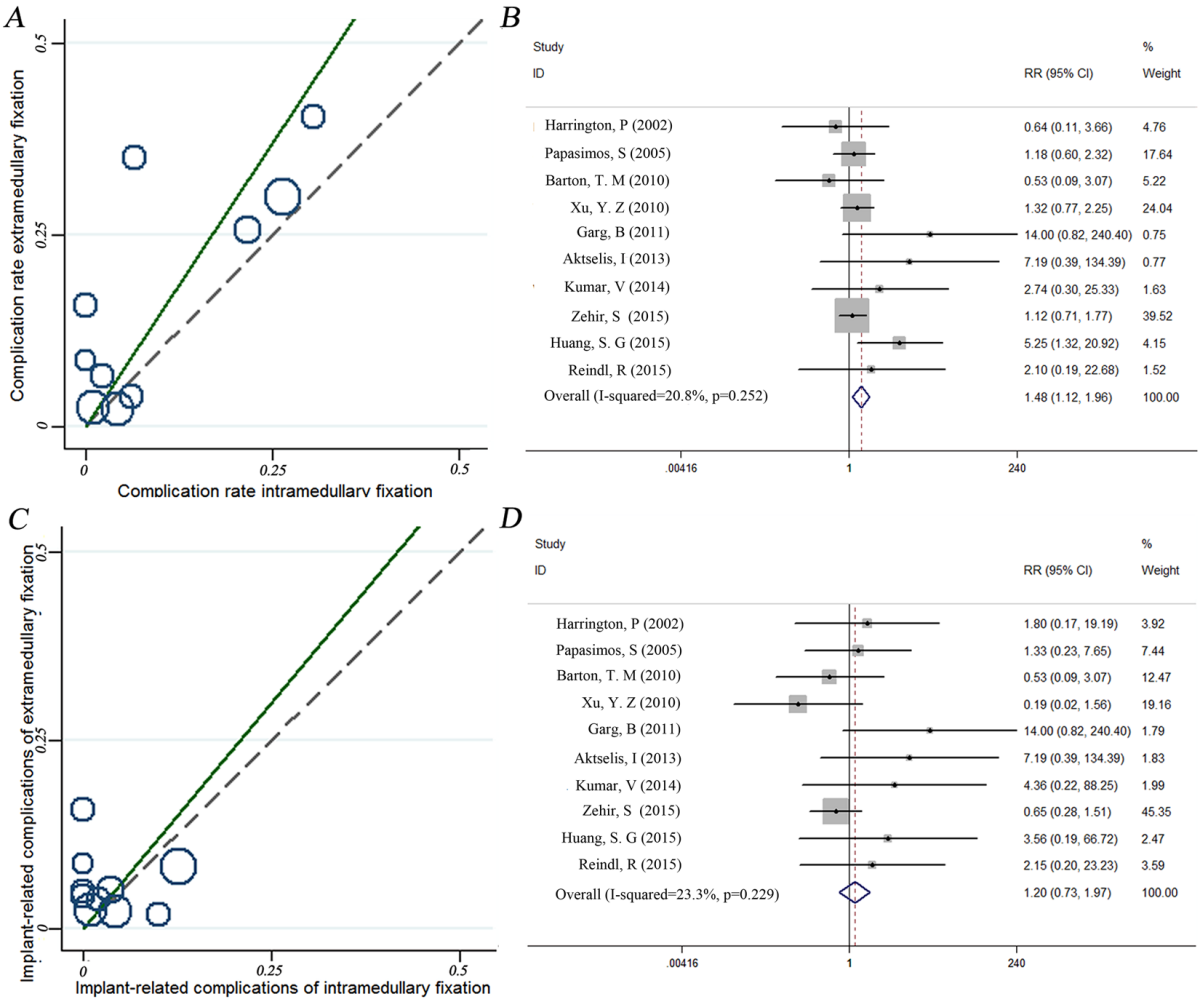
L’Abbé plot (**A**,**C**) and forest plot (**B**,**D**) for the meta-analysis of complication and implant-related complication rates between intramedullary nail and extramedullary fixation. L’Abbé plots of complications and implant-related complications indicated no obvious heterogeneity among the included studies, We observed significant differences in complication rates, but no significant difference in the incidence of implant-related complications between the two groups.

All 10 studies included data regarding complications, which mainly included deep vein thrombosis, wound infection, intra-operative complications, chest infection, pulmonary embolism, respiratory distress, mental disturbances, urinary tract infection, urinary retention, Femoral shaft fracture, Non-union, Cut-out, Migration of screw, Breakage of Implant were reported in the included studies and consisted mainly of femoral shaft fracture, non-union, cut-out, screw migration, implant breakage, and implant failure. The χ^2^ tests, I^2^ tests, and L’Abbé plots of complications indicated no obvious heterogeneity among the included studies (χ^2^ = 11.36, P = 0.252, I^2^ = 20.8%, Fig. 4A), so a fixed-effects model was used for the analysis. We observed significant differences in complication rates between the two groups (RR:1.48, 95% CI: 1.12–1.96, p = 0.006, Fig. 4B). We also carried out a subgroup analysis based on implant-related complications, such as Femoral shaft fracture, Non-union, Cut-out, Migration of screw, Breakage of Implant. No obvious statistical heterogeneity was observed among results for implant-related complications (Chi^2^ = 11.73, P = 0.229, I^2^ = 23.3%, Fig. 4C). Data pooled using a fixed-effects model indicated no significant difference in the incidence of implant-related complications between the two groups (RR:1.20, 95% CI: 0.73–1.97, p = 0.475, Fig. 4D).

### Publication bias

**Figure 5:**
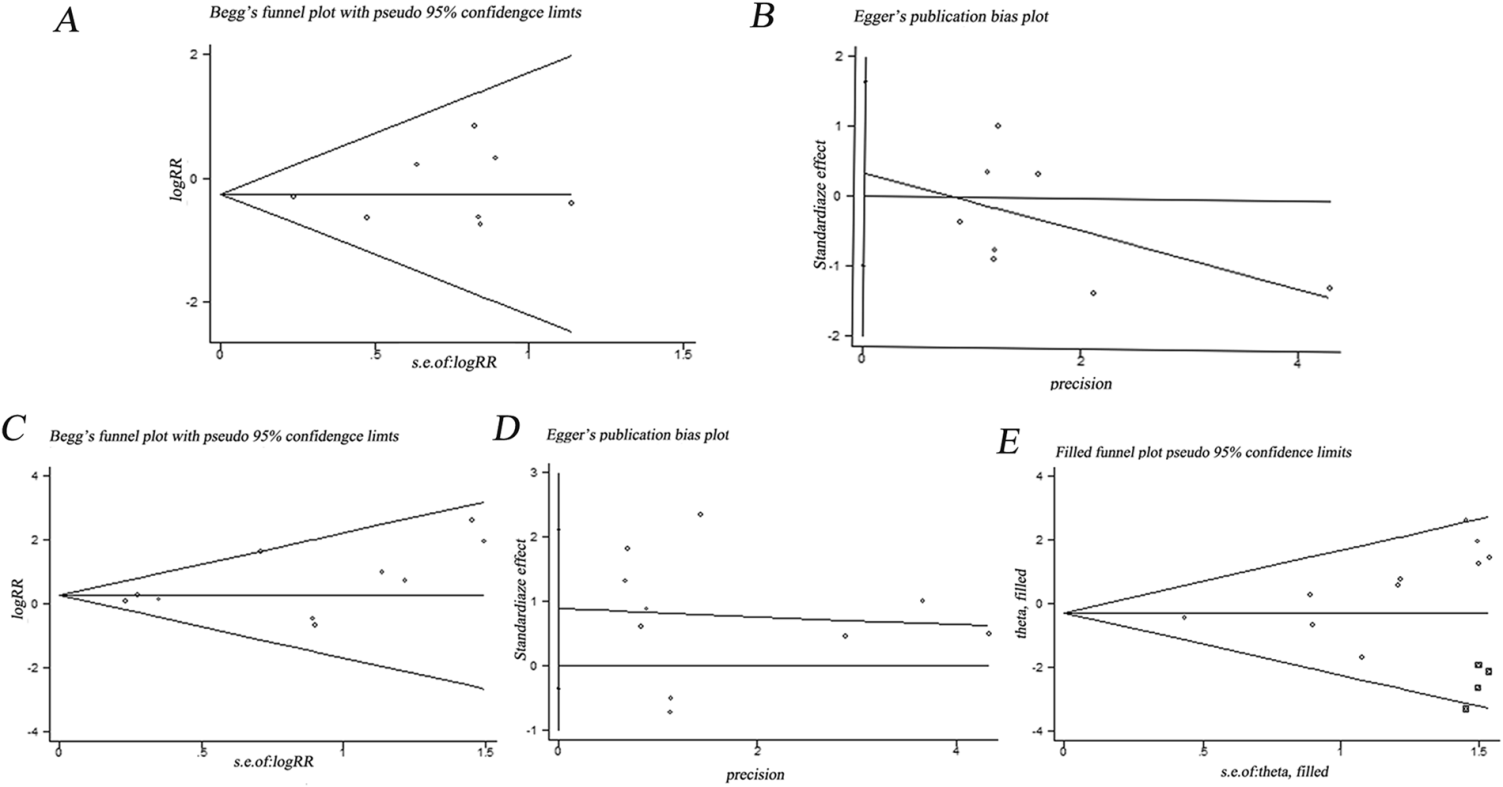
Funnel plot for publication bias. (**A**) Begg’s funnel plots and Egger’s test (**B**) of mortality. Begg’s funnel plots (**C**) and Egger’s test (**D**) of complications. (**E**) Duval and Tweedie nonparametric “trim and fill” method of accounting for publication bias for implant-related complications. There were no publication bias for mortality, complications and implant-related complications among these studies.

We assessed publication bias using Begg’s test and Egger’s test. The funnel plot for the meta-analysis of mortality for intramedullary nail versus extramedullary fixation was largely symmetric (P _Begg_ = 0.902, P _Egger_ = 0.567, Fig. 5A,B). Similar results were observed for complication rates (P_Begg_ = 0.210, P_Egger_ = 0.137, Fig. 5C,D). In addition, we performed the Duval and Tweedie nonparametric “trim and fill” method of accounting for publication bias in meta-analysis, which indicated no publication bias for implant-related complications among these studies (Fig. 5E). The pooled estimate of the fixed-effects model was −0.326 (95% CI: −0.857–0.204), while the pooled estimate of the random-effects model was −0.283 (95% CI: −1.061–0.496).

## Discussion

Unstable intertrochanteric fractures are difficult to manage^2^. Several fixation devices have been developed to overcome the difficulties encountered in the treatment of such fractures, including extramedullary (DHS, CHS, PFLCP, AMBI, SHS) and intramedullary (ITN, PFNA, GN) devices^28^. However, researchers have reported conflicting results regarding differences in mortality, functional outcomes, and complications between intramedullary nail and extramedullary fixation^11,14,29^. We therefore conducted a meta-analysis to determine whether evidence-based support for an optimal fixation device exists for unstable intertrochanteric fractures. In order to provide the best current evidence on the treatment of unstable intertrochanteric fractures, we included only studies that compared intramedullary nail and extramedullary devices. We identified 10 relevant randomized controlled trials and subsequently compared mortality rates, functional outcomes, and complications between patients treated with intramedullary nail and extramedullary fixation devices.

Johnell O concluded that hip fracture is a significant cause of morbidity and mortality worldwide^30^. Our study demonstrated no statistically significant difference in one-year mortality between intramedullary nail and extramedullary fixations and other research has shown that the advanced age and medical comorbidities led to a mortality rate of almost 10% within the first year after the fracture occurred^31^. Surgical management of unstable intertrochanteric fractures has evolved over the past few decades in a quest to improve functional outcomes in this patient population. when considering patients’ postoperative function of the two kinds of fixations, a literature review by I.B. Schipper suggested both intramedullary and extramedullary fixation offer clinical advantages for unstable trochanteric femoral fractures^4^. Another meta-analysis recommend the intramedullary nail technique for the treatment of unstable femoral intertrochanteric fractures due to better functional outcomes and reduced blood loss^28^. Our study pointed to exact p values from five studies indicated that functional outcomes were markedly better for intramedullary nail than extramedullary fixation (p = 0.0028). However, the lack of universally accepted functional scoring systems and low number of studies included in this analysis indicate that further investigation is required.

There are multiple factors that influences these two treatment options such as type of implant, type of fracture, patients age, co-mobidities, bone quality, time of treatment. Suitable implant selection according the type of fracture is an important factor to reduce the incidence of complication for unstable fracture in aged patients^32^. For the treatment of intertrochanteric fractures, intramedullary nail and extramedullary fixation, but each has advantages and disadvantages. On the one hand, Intramedullary fixation has multiple theoretical advantages for the treatment of unstable fracture patterns, such as biomechanical advantages, simple manipulation, small exposure, less complications, earlier mobilization. But they would be subject to the diameter of the medullary cavity and were inferior to extramedullary fixation in controlling rotational stability. Worse still, the endosteum blood supply was destroyed in the process of reaming. On the other hand, extramedullary fixation like the dynamic hip screw, whose advantage was interfragmental compression effect with a high union rate, and a minimally invasive technique was used to reduce soft tissue stripping and blood loss. But the extramedullary fixation had a higher incidence of varus collapse, medialization of the distal fragment and cut out of the femoral head screw in the treatment of unstable intertrochanteric fractures, which combined with lateral wall or posteromedial comminution, fractures with reverse obliquity patterns^33^. Honestly speaking, there was still no significant difference in the strength of fixation of stable and unstable infertrochanteric fractures between intramedullary nail and extramedullary fixation, although the intramedullary nail more rigid fixation^34^. Intertrochanteric fractures classified as unstable (AO/ASIF classification: 31-A2/31-A3), however, have a higher risk of complications and mechanical failure in comparison with stable fractures. Recent data have suggested that some unstable fracture patterns, such as reverse obliquity, and highly comminuted, could benefit from intramedullary nailing^35^. Comorbidities like osteoporosis may lead to technical problems during the procedure and complications sustained screw cut-out, loss of reduction, delayed union, malunion or nonunion, and various deformities of the femur, Because of the biome-chanical advantage and unique design (ITN and PFNA) for osteoporosis, the intramedullary nail appears to be a reliable implant in the management of intertrochanteric fracture in elderly patients with primary osteoporosis^36^. Higher operative time could result in more blood loss and higher infection rate, therefore, in order to reduce operative time, we had to choose to stick with their most-familiar implant system^37^.

More recent studies have reported little difference in complication rates and ambiguous clinical outcomes between intramedullary nail and open reduction/internal fixation (ORIF) surgical methods, making it difficult to determine the ideal implant due to risks and benefits associated with each device^8,38,39^. We observed a higher incidence of complications for extramedullary fixation than for intramedullary nail, which may be due to the biomechanical advantages of intramedullary fixation^34^. We also carried out a subgroup analysis based on implant-related complications, such as Femoral shaft fracture, Non-union, Cut-out, Migration of screw, Breakage of Implant and so on. Nevertheless, no significant differences in implant-related complications were observed between the groups. The main reasons responsible for the implant-related complications are such iatrogenic factors as biomechanically unsuitable position, unskillful surgical technique and improper post-operative instruction for functional exercise^40,41^.

The present meta-analysis, however, is limited in that few large-scale, multi-center RCTs specified for unstable femoral intertrochanteric fractures were included. Many trials included both stable and unstable fractures were not taken into account, only 10 published studies could be used for specific analysis of results in unstable fractures. Moreover, In our research, only five studies were included in this portion of the meta-analysis to evaluate the function. This fact limits the validity considerably. A significant difference considering all included studies was not possible. Besides, many trials failed to analyse results according to fracture type, patients age, co-mobidities, bone quality and time of treatment. As a result, Future large-scale studies should therefore aim to establish a universal standard for evaluating the efficacy of both treatments in this patient population. Similarly, evidence suggesting that patients treated with intramedullary nail experience better functional outcomes remains questionable, further supporting the need for a universal tool for the assessment of postoperative function. However, more conclusive evidence suggests that intramedullary nail is associated with fewer complications than extramedullary fixation, Therefore, the intramedullary nail method would be more reliable and should be encouraging for unstable intertrochanteric fractures.

## Materials and Methods

### Search strategy

We searched PubMed, Embase, Web of Science, and CBM databases using combinations of the following keywords: “unstable intertrochanteric fractures”, “intramedullary nail and extramedullary fixation”, “PFNA”, “PFN”, “Gamma nail”, “InterTan”, and “DHS”, “CHS”, “PFLCP”, randomized controlled trials” (last update on December 31, 2016). Reference lists for identified reports were also retrieved and reviewed for other potentially relevant studies. All studies were carefully evaluated for repeated data. Criteria used to define duplicate data included study period, hospital, treatment information, and any additional inclusion criteria.

### Inclusion and exclusion criteria

Studies that complied with the following criteria were eligible for inclusion in this study: (1) original design targeted toward only unstable intertrochanteric fractures; (2) prospective, randomized, multi-center design; (3) comparison of intramedullary nails and extramedullary fixation; (4) publication in English. Exclusion criteria were as follows: (1) type of literature specified as a “review”, “digest”, “talk”, “letter”, “commentary”, or “case report”; (2) cadaver or model-based studies; (3) duplicate or overlapping data; (4) retrospective design.

### Data extraction and quality assessment

Two authors independently extracted the data from all eligible articles, and any disagreements were resolved by discussion and consensus among the authors. Information retrieved for each study included author names, year of publication, original country, methods, number of patients, functional outcomes (clinical assessment scores) and associated *p* values, mortality, complications, implant-related complications. We also evaluated the potential for bias in all included studies. Evaluation criteria and methods followed the Cochrane Collaboration’s proposal. Statistical software Stata 12.0 (StataCorp LP, College Station, TX, USA) was used to assess the risk of bias.

### Statistical analysis

We evaluated differences in outcomes between intramedullary nail and extramedullary fixation by calculating the pooled relative risk (RR) and corresponding 95% confidence intervals (CI). Heterogeneity was assessed using chi-square and I-square tests. A fixed-effects model was used when there was no significant heterogeneity among the included studies (I^2^ ≤ 50%, P > 0.10). A random effects model was used when an obvious heterogeneity was observed among the included studies (I^2^ > 50%, P < 0.10). L’Abbé plots also demonstrated that there was no significant heterogeneity. Begg’s funnel plots and Egger’s test were used to assess the possibility of publication bias. Sensitivity analyses were also performed to assess the stability of the pooled effects. We performed statistical analysis with Stata version 12. A two-tailed P value less than 0.05 was considered statistically significant^42–44^.
